# Effects of Oxygenated Acids on Graphene Oxide: The Source of Oxygen-Containing Functional Group

**DOI:** 10.3389/fchem.2021.736954

**Published:** 2021-09-29

**Authors:** Xinghua Zhu, Yuanpu Xu, Zhibin Lu, Qunji Xue

**Affiliations:** State Key Laboratory of Solid Lubrication, Lanzhou Institute of Chemical Physics, Chinese Academy of Sciences, Lanzhou, China

**Keywords:** grapheme oxide, hummers method, oxygenated acid, oxygen-containing functional group, first principles

## Abstract

Graphene oxide is an important member of the graphene family which has a wide range of applications. The chemical method, especially the liquid phase method, is one of the most common and important methods for its preparation. However, the complex solution environment not only gives them rich structure, but also brings great challenges for its large-scale industrial synthesis. In order to better realize its industrial application, it is important to understand its structure, such as the source of oxygen-containing functional groups. Here we studied the contribution of four oxygenated acids to oxygen-containing functional groups in Hummers’ method using first principles. We found that the permanganic acid molecules that exist instantaneously due to energy fluctuations can be the source of oxygen-containing functional group. In addition, Stone-Wales defect have a certain effect on the formation of oxygen-containing functional groups, but this effect is not as good as that of solvation effect. This work provides a guide for exploring the source of oxygen-containing functional groups on graphene oxide.

## Introduction

Graphene oxide (GO), a very important member of the graphene family, has a wide range of applications in many fields, such as field effect transistors (Jin et al., 2009), sensors (Toda et al., 2015), transparent conductive films (Zheng et al., 2014), clean energy devices (Liang et al., 2009), etc., due to its rich variety and number of functional groups.

Numerous methods have been developed to synthesize GO, which can be categorized into eight (Brisebois and Siaj, 2020): 1) chemical (Brodie (Brodie, 1860), Staudenmaier (Staudenmaier, 1898), Hummers (Hummers and Offeman, 1958) method and their variant form such as Hofmann (Hofmann and König, 1937) and Tour (Marcano et al., 2010) method), 2) electrochemical (Ambrosi and Pumera, 2016; Pei et al., 2018), 3) microbial exfoliation for graphite (Zhu et al., 2013; Zhu et al., 2014; Liu et al., 2015), oxidative chemical for 4) 3D-carbon structures (Luo et al., 2009; Zhang et al., 2016) and 5) 2D-graphene (Nourbakhsh et al., 2010; Zhao et al., 2012), 6) chemical vapor deposition (CVD) (López et al., 2009; Huang et al., 2013), 7) hydrothermal methods for carbohydrate (Tang et al., 2012; Krishnan et al., 2014) and 8) thermal decomposition methods for organic matter rich in carbon (Prías-Barragán et al., 2016; Goswami et al., 2017). Nowadays, many other methods have also been developed for the preparation of GO. But in general, it is usually prepared by chemical methods (Zhu et al., 2010).

These methods are used to react the graphene with strong oxidising solution, such as potassium chlorate (KClO_3_) with nitric acid (HNO_3_) (Brodie and Staudenmaier methods), combination of potassium permanganate (KMnO_4_) and sulfuric acid (H_2_SO_4_) (Hummers’ method), etc., so that there are a large number of different functional groups on the surface and on the edge of the graphene to get the GO. Different methods result in different structures (Brisebois and Siaj, 2020) due to complexity of aqueous solutions, which gives it variable performance and wide application. Until now, the structure of GO remains controversial although there are many theoretical models that can partially explain its structure, such as Lerf–Klinowski model (He et al., 1996; Lerf et al., 1997; He et al., 1998; Lerf et al., 1998), Dékány model (Szabó et al., 2005; Szabó et al., 2006; Gao et al., 2009), etc. Previous studies have shown that GO structure is closely related to a variety of factors such as the type and quantity of the solution (Dimiev et al., 2012; Eigler et al., 2013; Eng et al., 2013), structural holes (Erickson et al., 2010), radical reaction (Collins et al., 2011; Dimiev et al., 2012; Eigler et al., 2013; Yang et al., 2014), etc. Based on these, a large number of experimental studies have been carried out in detail with great success in terms of carbon and oxygen content (Erickson et al., 2010; Collins et al., 2011; Dimiev et al., 2012; Eigler et al., 2013; Yang et al., 2014; Brisebois et al., 2016), functional group types (He et al., 1996; Lerf et al., 1997; Lerf et al., 1998; Collins et al., 2011), different types and proportions of solutions, the microscopic process of exfoliation (Zhang et al., 2009; Tang et al., 2015; Betancur et al., 2018), etc. Meanwhile, the fact that oxygen functional groups are introduced (Skaltsas et al., 2013; Ma et al., 2018) through topological defects (Bracamonte et al., 2014) by ultrasonic treatment and solvent molecules has also been clarified. All of these provide a solid foundation for the production and application of GO.

Nevertheless, the technology to produce GO is still being studied, which plays a decisive role in the large-scale and stable production of GO and other graphene derivatives such as reduced graphene oxide (rGO). The source of oxygen-containing functional group, especially that of oxygen, is one of the most important question because these groups can greatly affect the structure and properties of GO (Johari and Shenoy, 2011; Das et al., 2013). Oxygen is usually thought to come from one component in solution such as H_2_O_2_ (Yang et al., 2014) and KClO_3_ (Dreyer et al., 2010), but other study have shown that it can also come from air (Skaltsas et al., 2013). In this process, water can enhance the degree of oxidation and regulate the content of hydroxyl and epoxy groups (Chen et al., 2016). One hypothesis is that most of oxygen comes from certain components of solvent and a little from air. However, a question arises as to which solvent the oxygen is most likely to come from because many oxygen-containing solvents, such as KMnO_4_, KClO_3_, HNO_3_, H_2_SO_4_, etc., are used in these methods. In addition, the relationship between the introduction of functional groups and the type of defects is also a very important question. Whether the defect is selective of functional groups, for instance, although the fact that epoxy functional groups can be introduced through topological defects caused by ultrasonic treatment and solvent molecules has been clarified (Skaltsas et al., 2013; Bracamonte et al., 2014; Ma et al., 2018). In the solution environment, ultrasonication can also improve the oxidation level of GO by the mechanical shear force and shock wave generated by the collapse of cavitation bubble (Qi et al., 2014) during the ultrasound process. These issues are critical to the structure and properties of GO.

Solving these problems requires a deep understanding of the relationship between graphene and these oxygenated solvents. Here, we investigated the relationship between some oxygenated acid (including sulfuric acid (H_2_SO_4_), nitric acid (HNO_3_), permanganic acid (HMnO_4_), chloric acid (HClO_3_) and their mixture acid) present in Hummers’ method and non-defect and Stone-Wales defect graphene using first principles. We looked at the role of not only individual components but mixtures of components in order to explore the contribution of each component. We also consider the perturbation of these components by water molecules. Density of states (DOS), adsorption energy, bond length, charge transfer and overlapping population were mainly used to study the relationship between molecules and non-defect and Stone-Wales defect graphene. This work provides a guide for exploring the source of oxygen-containing functional groups on graphene oxide.

## Calculation Details

This work uses the Cambridge Sequential Total Energy Package (CASTEP) (Hohenberg and Kohn, 1964; Kohn and Sham, 1965; Vanderbilt, 1990; Clark et al., 2005), which is based on the density functional theory (DFT). We use the Perdew-Burke-Ernzerhof (PBE) that belongs to the General Gradient Approximation (GGA) function to describe the exchange-correlation effect (Perdew et al., 1996). The Ultrasoft pseudopotential is used to describe the real potential of electrons (Vanderbilt, 1990). All Bands/EDFT electronic minimization scheme is applied in Self-Consistent Field (SCF) convergence (Marzari et al., 1997). Self-consistent scheme (Neugebauer and Scheffler, 1992) and grimme method (Rydberg et al., 2003; Grimme, 2011) are used for dipole-dipole interactions and DFT-D2 correction, respectively. Bond population based on Mulliken’s work (Mulliken, 1955) and charge analysis derived from Hirshfeld’s work (Hirshfeld, 1977) are applied to analyze the bond strength and the direction of charge transfer, respectively.

In this study, the cut-off energy is set to 600 eV. Vacuum space of 15 Å is applied to avoid the effect of periodicity on the Z axis. Convergence threshold of 1 × 10^–6^ eV/atom is applied in SCF. In this work, two sizes of substrate model were used for the calculation. For non-defect graphene, the model has 32 atoms, and it has 50 atoms when it contains Stone-Wales defect. K-point grid of 5 × 5 × 1 is applied to compute these models. It was raised to 7 × 7 × 1 when DOS is calculated. All models are optimized so that these forces are less than 0.02 eV/Å.

## Results and Discussion

In this study, we set up two types of comparison groups. One consisted of a single component, and the other one consisted of multiple components. For the former, it involves the reaction of H_2_SO_4_, HNO_3_, HMnO_4_ and HClO_3_ with non-defect and Stone-Wales defect graphene, respectively. The latter, on the other hand, includes four types: mixture of H_2_SO_4_ and HNO_3_, that of H_2_SO_4_ and HMnO_4_, that of HNO_3_ and HMnO_4_, and that of all three.

### Reaction of Single Component With Graphene

**FIGURE 1:**
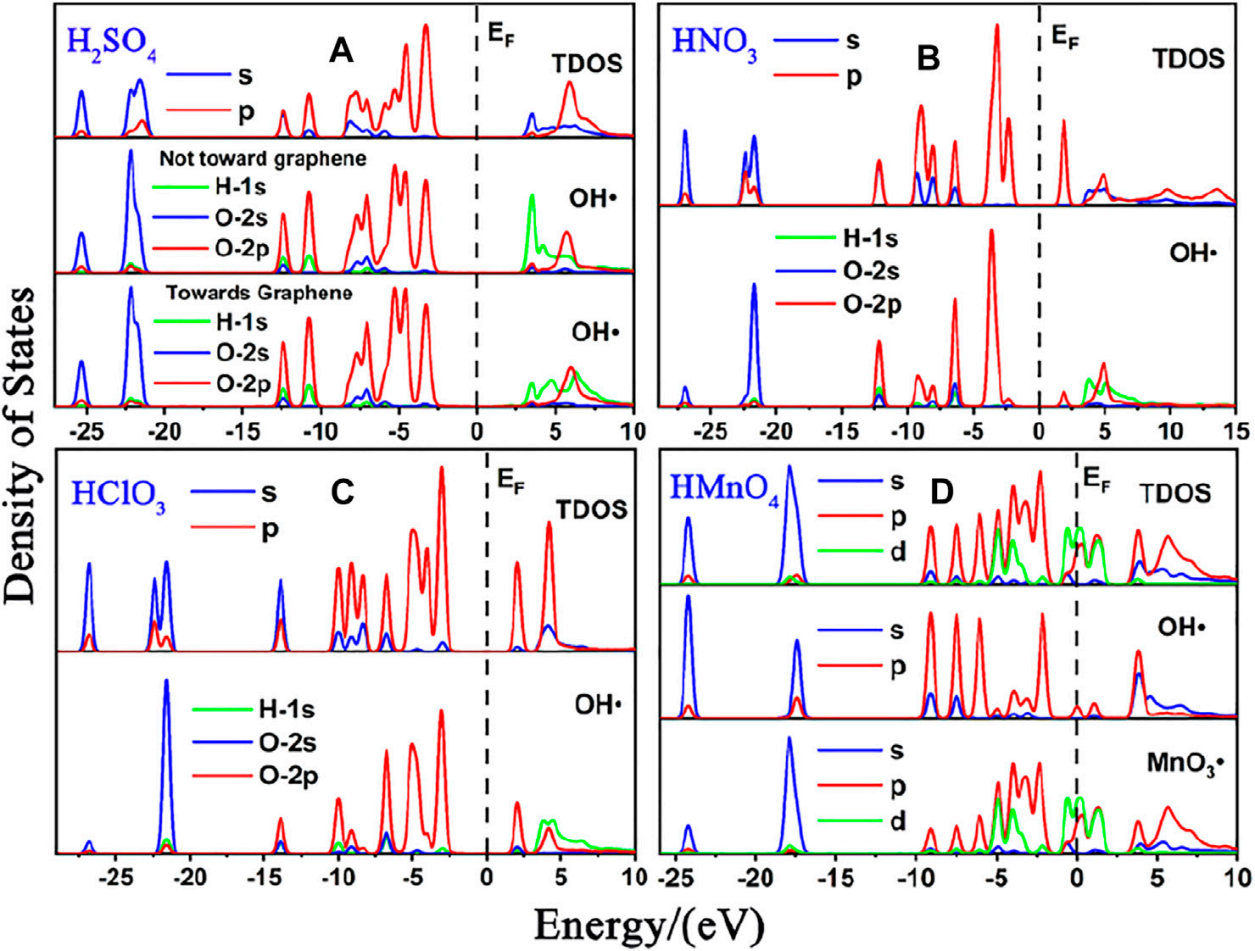
DOS of single-component acid molecules reacting with the non-defective graphene in the absence of water. **(A)**, **(B)**, **(C)**, and **(D)** are the DOS of H_2_SO_4_, HNO_3_, HClO_3_ and HMnO_4_, respectively. In each diagram, the top represents the total density of states (TDOS), and the bottom represents the local density of states (LDOS) of OH•. Particularly, there is also the LDOS of MnO_3_• at the bottom of **(D)**.

**FIGURE 2:**
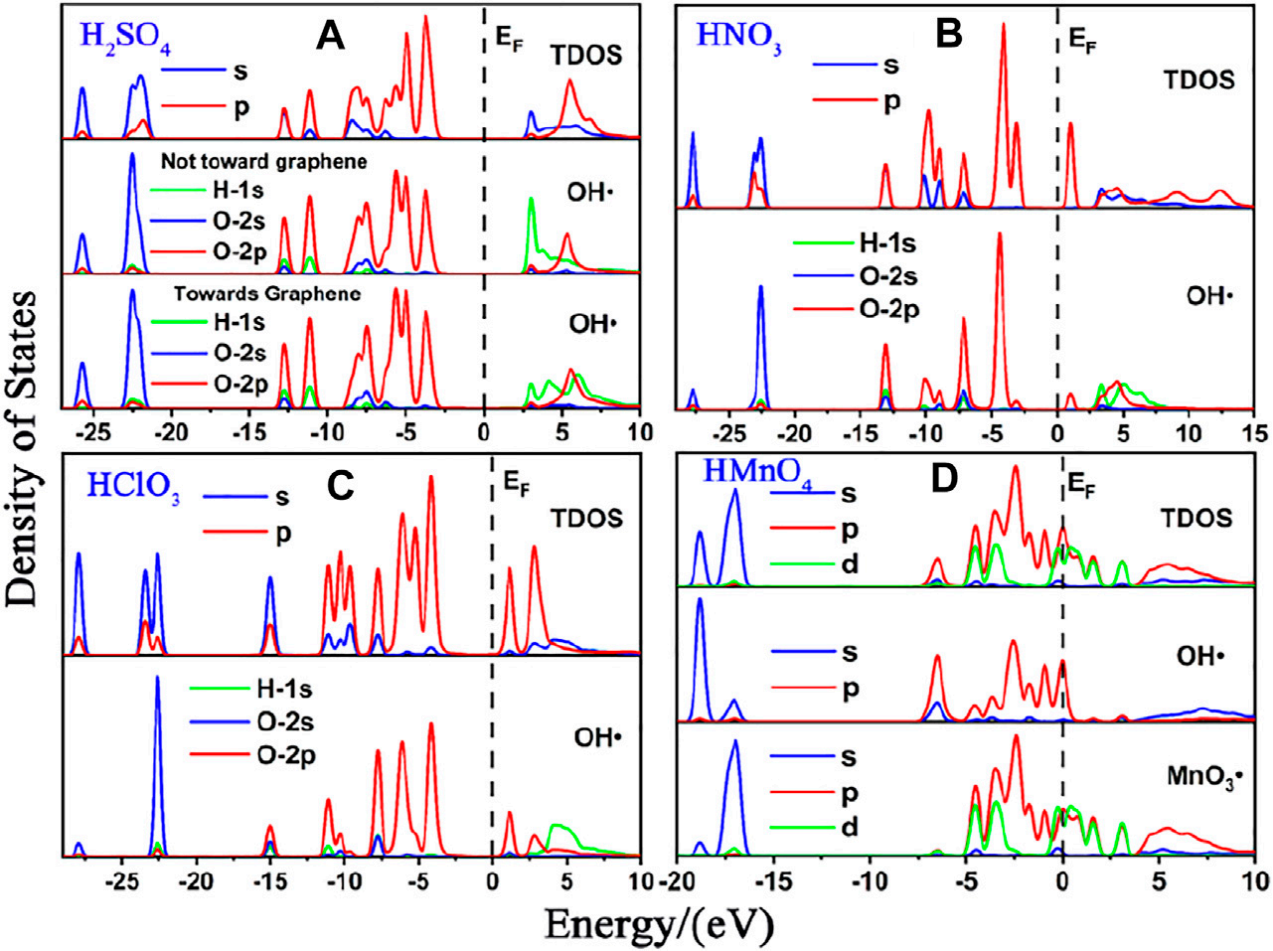
DOS of single-component acid molecules reacting with the Stone-Wales defect graphene in the absence of water. **(A)**, **(B)**, **(C)**, and **(D)** are the DOS of H_2_SO_4_, HNO_3_, HClO_3_ and HMnO_4_, respectively. In each diagram, the top represents the TDOS, and the bottom represents the LDOS of OH•. Particularly, there is also the LDOS of MnO_3_• at the bottom of **(D)**.

**FIGURE 3:**
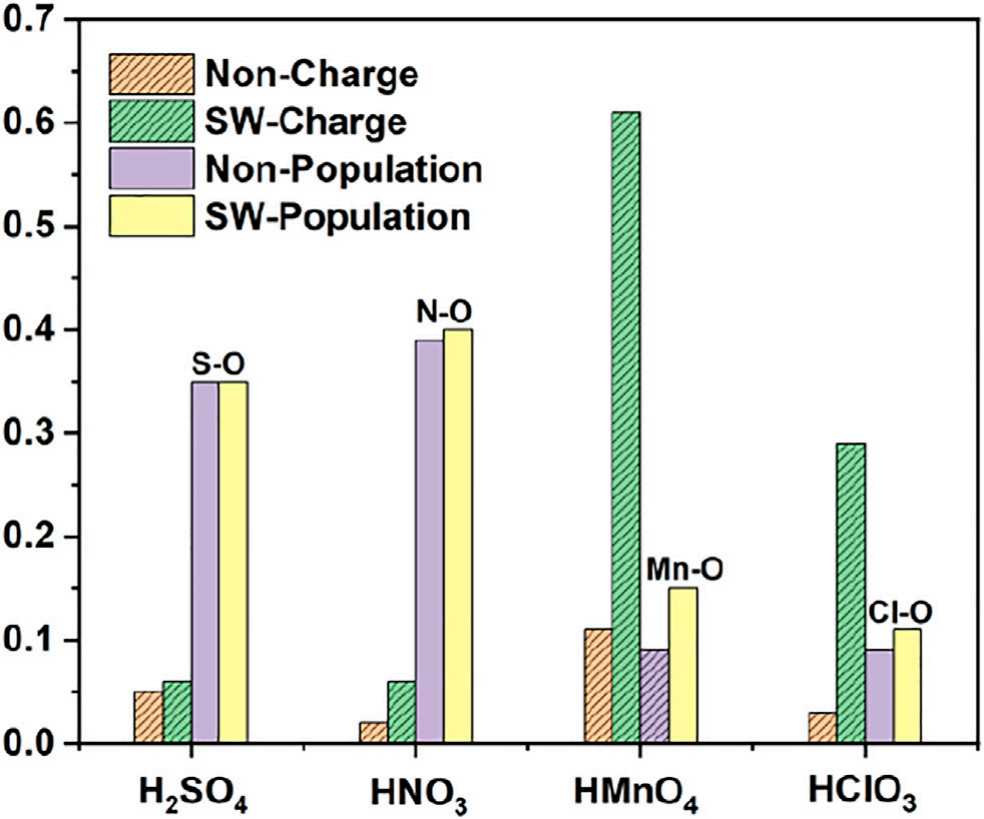
Charge and bond population of single-component acid molecules reacting with the non-defect and Stone-Wales defect graphene in the absence of water. The shadow indicates that this value is negative.

We first studied the case of single-component without water. DOS of single-component acid molecules reacting with the non-defective and Stone-Wales defect graphene are shown in Figures 1, 2. The calculation results (including the adsorption energy (E_ads_), bond length, charge transfer (△Q) and overlapping population) are shown in Figure 3, Table 1.

**TABLE 1:** Reaction of single component on the surface of graphene without water.

	Defect	Dissociation	E_ads_/(eV)	Bond length
H_2_SO_4_	No	No	−0.3648	S-O: 1.59
HNO_3_	No	No	0.0088	N-O: 1.43
HMnO_4_	No	Yes	−0.2206	Mn-O: 2.80
HClO_3_	No	No	−0.1768	Cl-O: 1.70
H_2_SO_4_	SW	No	−0.4102	S-O: 1.59
HNO_3_	SW	No	−0.2455	N-O: 1.42
HMnO_4_	SW	No	−2.8110	Mn-O: 2.10
HClO_3_	SW	No	−0.4797	Cl-O: 1.68

There is only weak physical adsorption between these molecules and non-defect or Stone-Wales defect graphene, as is shown in Figure 1 and Table 1. The amount of charge transfer between these molecules and graphene is also small. These charges are transferred from graphene to hydrogen atoms of the acid molecules, hence the DOS of OH• at the right side of the Fermi energy level is diffused to some extent, especially in the 1s orbital of hydrogen atom, as shown in Figures 1A,B,C and Figures 2A,B,C. This can be more clearly shown by the DOS of H_2_SO_4_. As shown in Figure 2A, the H_2_SO_4_ molecule contains two OH•, one of which is close to the graphene and the other is far away from it. Therefore, the one close to the graphene will experience energy dispersion due to electron injection, while the other will remain relatively local.

But for HMnO_4_, the results are different when it reacts with non-defect and Stone-Wales defect graphene. The molecule will dissociate to form MnO_3_• and OH• when it reacts with non-defective graphene. In this case, MnO_3_• and OH• are combined together to form a group through strong Coulomb interaction that takes place between two oxygen atoms (population of O-O bond: 0.23, binding energy: −2.9152 eV). While this dissociation did not occur between HMnO_4_ and Stone-Wales defects. The manganese atom and the oxygen atom in OH• are still bound together by a relatively strong ionic bond (population of Mn-O bond: 0.15). In the case of HMnO_4_ reacting with non-defective graphene, therefore, there are many energy levels with strong localization in the DOS of MnO_3_• and OH• around the Fermi level, especially in the range of −10∼−5 eV, which are mainly contributed by the 2p orbital of oxygen atom, seeing in Figure 2D. Meanwhile, the energy values corresponding to these levels are consistent, which also indicates that there is a strong Coulomb interaction between MnO_3_• and OH•. Besides, the level to the right of Fermi level of OH• formed by dissociation still has a high localization, indicating that this part has fallen off from the molecule. In the other case, levels near the Fermi level are non-local because it does not dissociate. In other words, there not have levels with strong localization near the Fermi level. The 1s orbital of hydrogen atom at the right of Fermi level becomes diffused due to the charge received from graphene because the bond between HMnO_4_ and Stone-Wales defect graphene is strong chemisorption, seeing in Figure 2D, Figure 3, Table 1. Direction of charge transfer is from graphene to HMnO_4_. This phenomenon indicates that the Stone-Wales defect does have selectivity for functional groups, as previous study have shown that it is conducive to the introduction of epoxy groups (Bracamonte et al., 2014). During the ultrasound process, ultrasonication can also improve the oxidation level (Qi et al., 2014).

**FIGURE 4:**
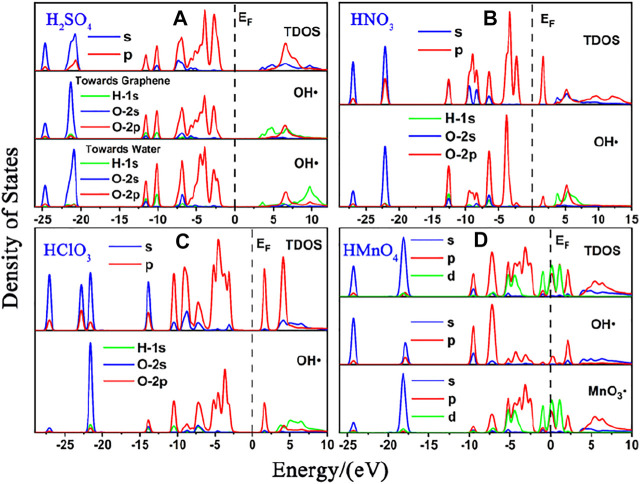
DOS of single-component acid molecules reacting with the non-defective graphene in aqueous condition. **(A)**, **(B)**, **(C)**, and **(D)** are the DOS of H_2_SO_4_, HNO_3_, HClO_3_ and HMnO_4_, respectively. In each diagram, the top represents the TDOS, and the bottom represents the LDOS of OH•. Particularly, there is also the LDOS of MnO_3_• at the bottom of **(D)**.

**FIGURE 5:**
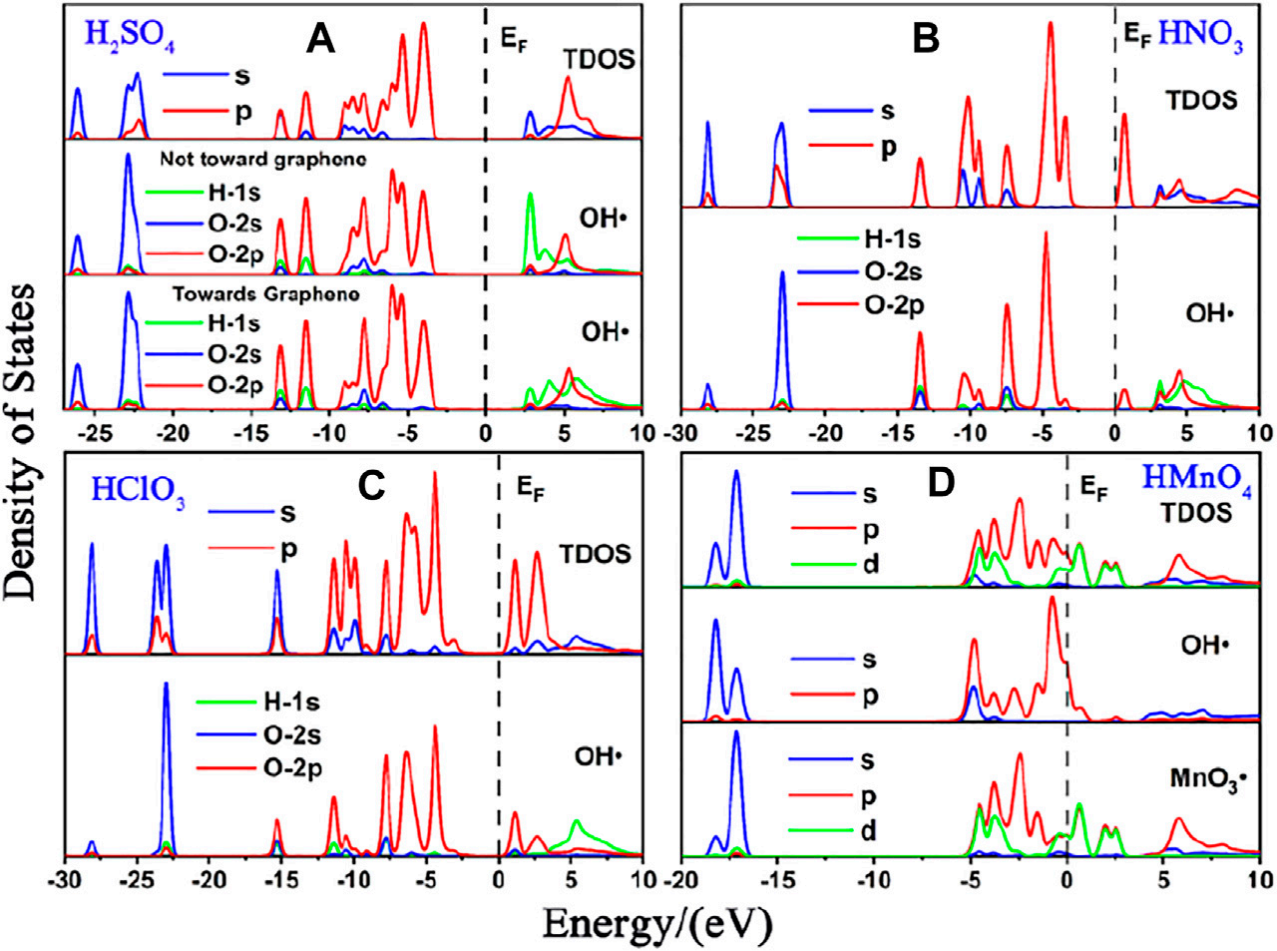
DOS of single-component acid molecules reacting with the Stone-Wales defect graphene in aqueous condition. **(A)**, **(B)**, **(C)**, and **(D)** are the DOS of H_2_SO_4_, HNO_3_, HClO_3_ and HMnO_4_, respectively. In each diagram, the top represents the TDOS, and the bottom represents the LDOS of OH•. Particularly, there is also the LDOS of MnO_3_• at the bottom of **(D)**.

**FIGURE 6:**
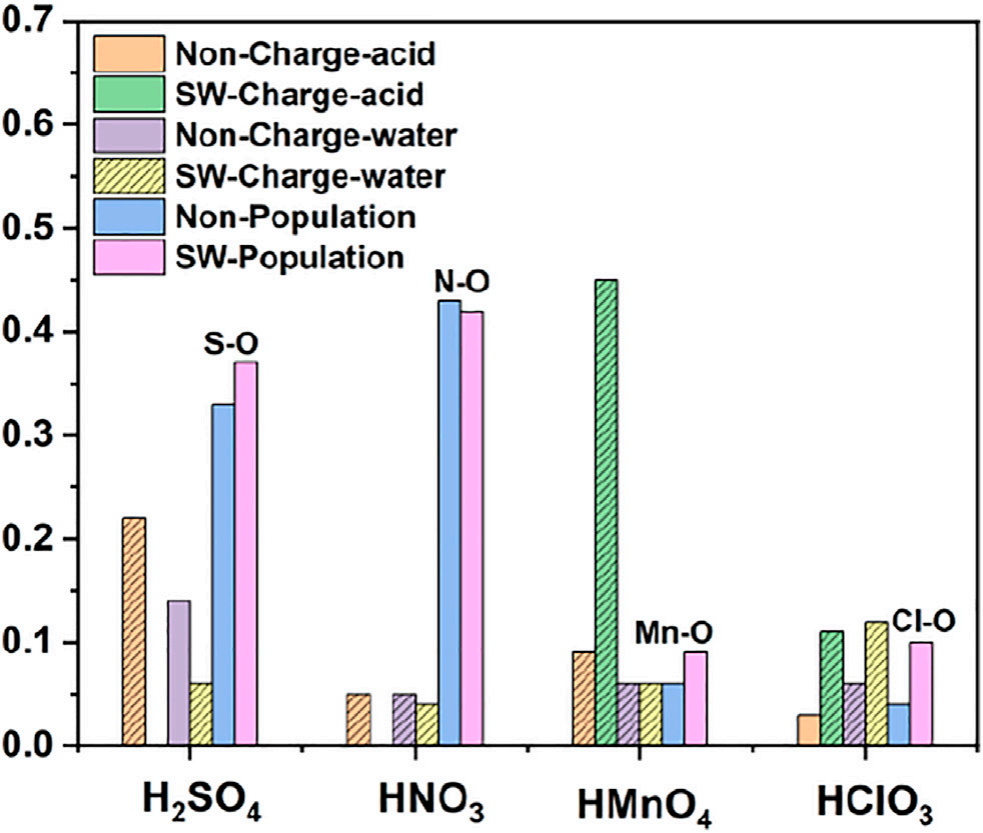
Charge and bond population of single-component acid molecules reacting with the non-defect and Stone-Wales defect graphene in aqueous condition. The shadow indicates that this value is negative.

Perturbations of water molecules are ignored in the above models. Therefore, we considered the effect of water molecules. DOS of these are shown in Figures 4, 5. The calculated data are shown in Figure 6, Table 2 and Table S2.

**TABLE 2:** Reaction of single component on the surface of graphene with water.

	Defect	Dissociation	E_ads_/(eV)	Bond length
H_2_SO_4_	No	No	−0.4897	S-O: 1.61
HNO_3_	No	No	−0.3252	N-O: 1.39
HMnO_4_	No	No	−0.5695	Mn-O: 2.04
HClO_3_	No	No	−0.3918	Cl-O: 1.74
H_2_SO_4_	SW	No	−0.4979	S-O: 1.58
HNO_3_	SW	No	−0.3577	N-O: 1.41
HMnO_4_	SW	Yes	−1.1313	Mn-O: 2.58
HClO_3_	SW	No	2.1108	Cl-O: 1.65

As in the case of non-aqueous molecules, both the non-defect graphene and Stone-Wales defect graphene have only weak physical adsorption between H_2_SO_4_, HNO_3_ and HClO_3_ and graphene. The amount of charge transferred from graphene to molecules is small, as is shown in Figure 6. These are represented by the DOS in which the OH• in the molecule is diffused to some extent to the right of the Fermi level. This occurs primarily in the 1s orbital of hydrogen atoms, seeing in Figures 4A–C and Figures 5A–C.

The dissociation of HMnO_4_ is opposite of that without water. In the case of water-containing molecules, the reaction between HMnO_4_ and non-defective graphene only produces a tendency to dissociate. Among them, the OH• on the molecule rotates at a large angle toward the neighboring oxygen atom. Only chemisorption exists between HMnO_4_ and graphene without defects. However, the chemical bond between manganese and OH• is greatly weakened (bond population of Mn-O: 0.06) although the HMnO_4_ does not dissociate, indicating that OH• tends to detach from the molecule. Therefore, as shown in Figure 4D, some strongly localized energy levels contributed by the 2p orbital of oxygen atom appear in both MnO_3_• and OH• in the range of −10∼−5 eV. The energy values corresponding to these energy levels are consistent, indicating that there is still a strong bond between MnO_3_• and OH•. By contrast, HMnO_4_ dissociates on Stone-Wales defect graphene to form MnO_3_• and OH•. But MnO_3_• and OH• did not combine together to form a neutral group by strong coulomb interaction like the case of HMnO_4_ disassociate on the defection-free graphene. These two parts are relatively independent. Therefore, there are very few energy levels with similar energy values near the Fermi level between MnO_3_• and OH•, seeing in Figure 5D. In addition, the OH• in both cases becomes diffuse at the right side of Fermi level because of the charge received from graphene, as shown in Figure 4D and Figure 5D. This phenomenon indicates that water contributes to the formation of oxygen-containing functional groups, as previous study (Chen et al., 2016) have shown. In other words, solvation effect contributes to the formation of functional groups. This is stronger than that of topological defects although Stone-Wales defects have selectivity for functional groups.

### Reaction of Mixed Components With Graphene

In the preparation of GO, a mixture of solvents is usually used rather than a single-component reagent. Therefore, on the basis of *Reaction of Single Component With Graphene*, this section further studied the reaction between the mixed acid molecules and graphene. In this section, the reactions between H_2_SO_4_, HMnO_4_ and HClO_3_ and graphene are mainly calculated, considering the types of reagents needed for the preparation of GO. In the calculation, two contrast groups were set up for the reaction with non-defective graphene and Stone-Wales defect graphene. Each of the above categories included the following three groups: H_2_SO_4_ and HMnO_4_, HNO_3_ and HMnO_4_, H_2_SO_4_ and HNO_3_ and HMnO_4_.

**FIGURE 7:**
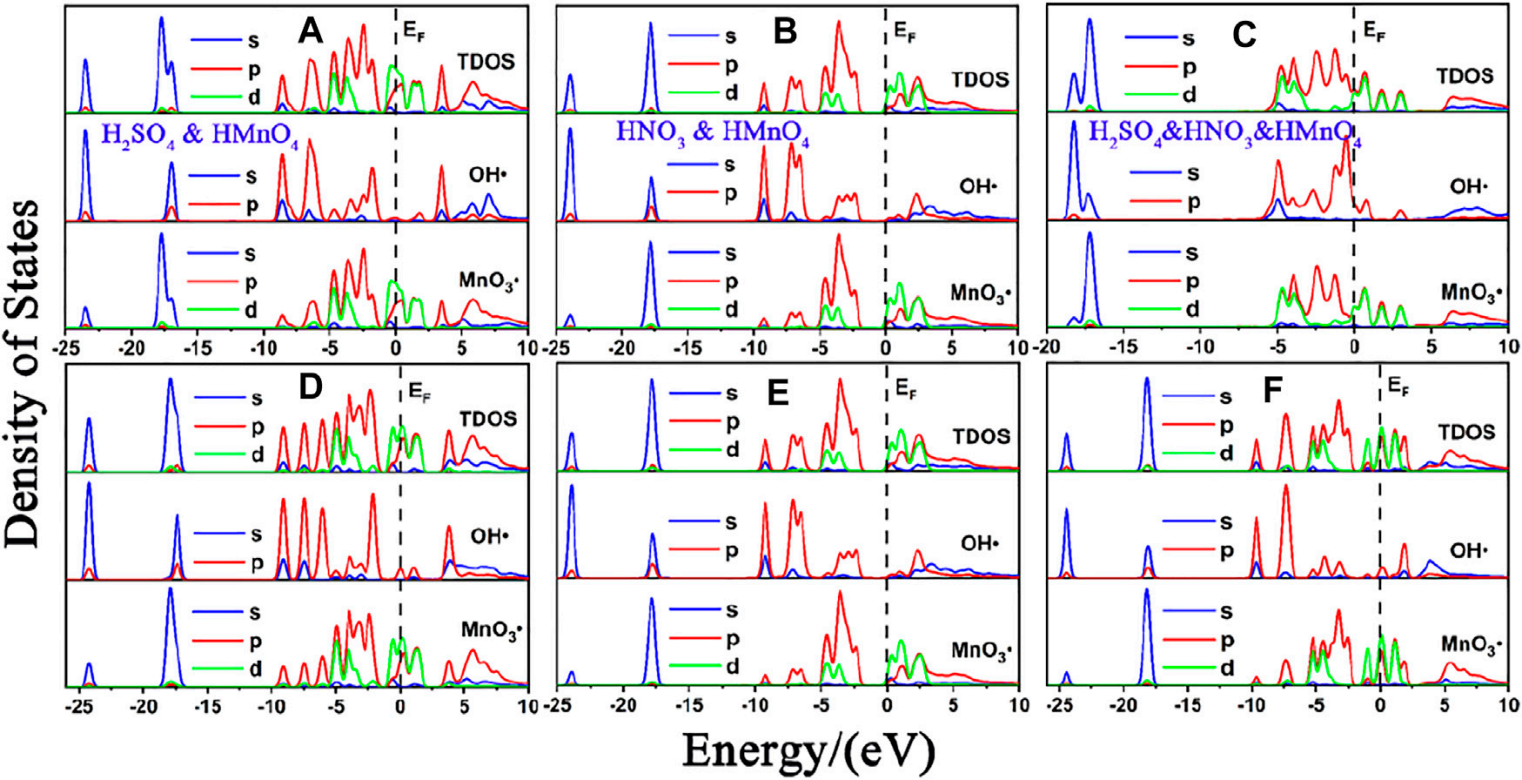
DOS of mix-component acid molecules in the absence of water. **(A)**, **(B)** and **(C)** are the DOS of HMnO_4_ in the case of H_2_SO_4_ and HMnO_4_, HNO_3_ and HMnO_4_ and H_2_SO_4_ and HNO_3_ and HMnO_4_ on non-defect graphene, respectively. **(D)**, **(E)** and **(F)** are the DOS of HMnO_4_ in the case of H_2_SO_4_ and HMnO_4_, HNO_3_ and HMnO_4_ and H_2_SO_4_ and HNO_3_ and HMnO_4_ on Stone-Wales defect graphene, respectively. In each diagram, the top represents the TDOS, the middle represents the LDOS pf MnO_3_• and the bottom represents the LDOS of OH•.

**FIGURE 8:**
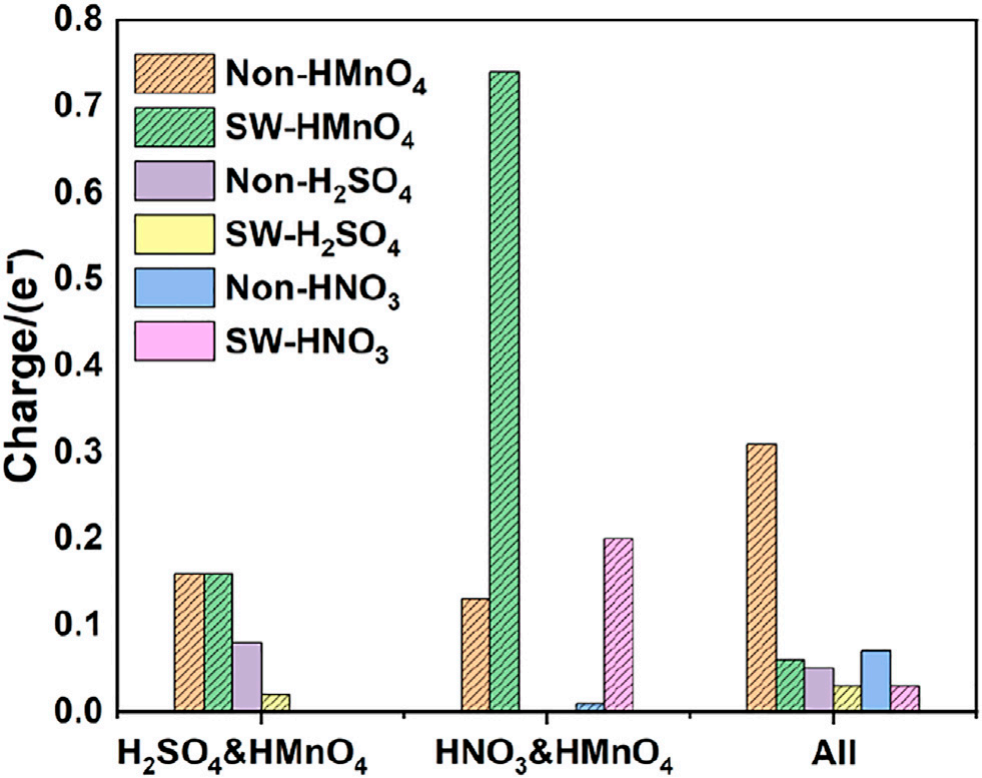
Charge of HMnO_4_ in the mix-component in the absence of water. The shadow indicates that this value is negative.

DOS of these are shown in Figure 7. The calculated data are shown in Figure 8, Table 3.

**TABLE 3:** Reaction of mixed components on the surface of graphene without water.

	Defect	Dissociation	E_ads_/(eV)
H_2_SO_4_ and HMnO_4_	No	Yes	−0.4361
HNO_3_ and HMnO_4_	No	Yes	−0.4209
All	No	No	−0.7780
H_2_SO_4_ and HMnO_4_	SW	Yes	−0.6074
HNO_3_ and HMnO_4_	SW	Yes	−0.5941
All	SW	Yes	−0.7330

Similar to the case of single components, there is only weak physical adsorption between sulfuric and nitric acid molecules and graphene in the mixed condition, whether graphene is perfect or with Stone-Wales defects. H_2_SO_4_ and HNO_3_ molecules will not dissociate form OH•, but their presence will lead to the occurrence of solvation effect. It can promote the dissociation of HMnO_4_ to form MnO_3_• and OH•, as shown in Figures 7A,B,D–F. What is unique in these cases is the reaction between a mixture of three acids and the non-defective graphene in which HMnO_4_ is not dissociated. These phenomena further prove that the effect of solvation is stronger than that of topological defects although the latter has selectivity for functional groups.

**FIGURE 9:**
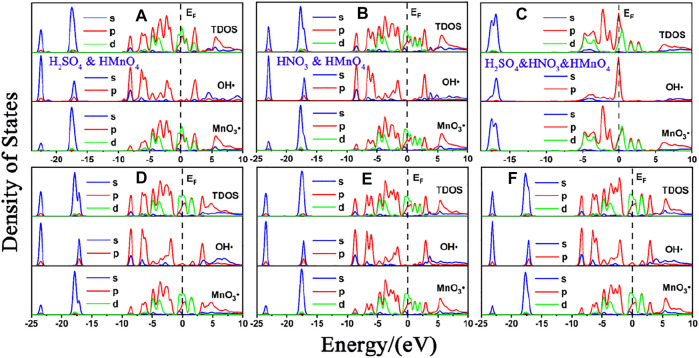
DOS of mix-component acid molecules in aqueous condition. **(A)**, **(B)** and **(C)** are the DOS of HMnO_4_ in the case of H_2_SO_4_ and HMnO_4_, HNO_3_ and HMnO_4_ and H_2_SO_4_ and HNO_3_ and HMnO_4_ on non-defect graphene, respectively. **(D)**, **(E)** and **(F)** are the DOS of HMnO_4_ in the case of H_2_SO_4_ and HMnO_4_, HNO_3_ and HMnO_4_ and H_2_SO_4_ and HNO_3_ and HMnO_4_ on Stone-Wales defect graphene, respectively. In each diagram, the top represents the TDOS, the middle represents the LDOS pf MnO_3_• and the bottom represents the LDOS of OH•.

**FIGURE 10:**
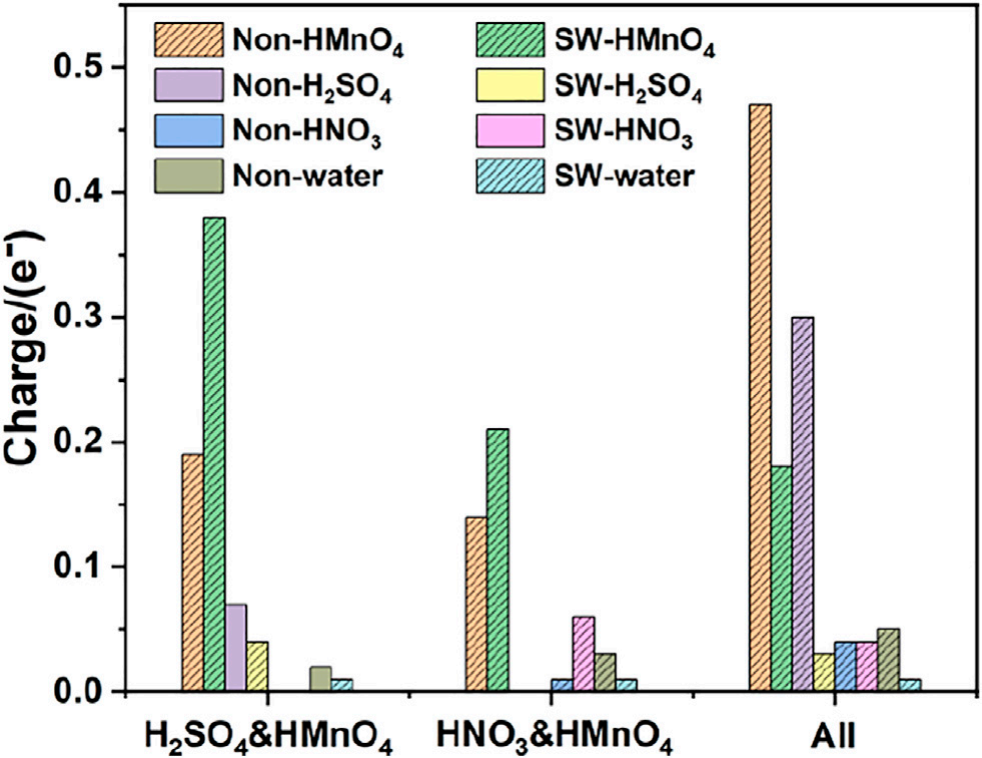
Charge of HMnO_4_ in the mix-component in aqueous condition. The shadow indicates that this value is negative.

In order to be closer to the experimental content of the preparation of GO, water molecules were added to the above model to further confirm the above conclusions. DOS of these are shown in Figure 9. The calculated data are shown in Figure 10, Table 4.

**TABLE 4:** Reaction of mixed components on the surface of graphene with water.

	Defect	Dissociation	E_ads_/(eV)
H_2_SO_4_ and HMnO_4_	No	Yes	−0.3710
HNO_3_ and HMnO_4_	No	Yes	−0.4923
All	No	Yes	−0.8583
H_2_SO_4_ and HMnO_4_	SW	Yes	−1.6667
HNO_3_ and HMnO_4_	SW	Yes	−0.6199
All	SW	Yes	−0.7761

Consistently with the previous results, there is only weak physical adsorption due to van der Waals forces between H_2_SO_4_ and HNO_3_ molecules and graphene under the condition of mixed acid, no matter for perfect or Stone-Wales defect graphene. The amount of charge transfer between these molecules and graphene is very small, seeing in Figure 10. The presence of water, H_2_SO_4_, and HNO_3_ molecules leads to solvation effect that dissociates HMnO_4_ to form OH•. Meanwhile, this effect is enhanced due to the presence of multiple components. In all the models, HMnO_4_ disassociates to produce OH•. These OH• can attach to the surface of graphene and become the source of hydroxyl groups in GO. They can also react with the oxygen present in the solution or air to form carboxyl groups. They are able to react with other components to generate further dissociation and become the source of the epoxy functional groups, too. These phenomena strongly prove that the effect of solvation is stronger than that of topological defects although Stone-Wales defects have selectivity for functional groups. Therefore, based on the above situation, it can be considered that the HMnO_4_ molecules generated instantaneously due to energy fluctuations in the solution environment can become the source of oxygen-containing functional groups on GO.

## Conclusion

In summary, in the solution environment where GO is prepared, the HMnO_4_ molecules that exist instantaneously due to energy fluctuations can disassociate to form OH•, and OH• can be a source of hydroxyl groups in GO. They can also react with the oxygen present in the solution or air to form carboxyl groups. They are able to react with other components to generate further dissociation and become the source of the epoxy functional groups, too. In this process, the Stone-Wales defect plays a certain role in the formation of functional groups, but this role is far less than that of the solvation effect. In complex solution environment, many particles will interact and influence each other. Among them, non-permanganate related components from graphene and solution can constantly transfer charge to permanganate. The mixed components can make the reaction proceed in the direction of dissociation, so as to promote the dissociation of permanganate to form OH•. This work provides a guide for exploring the source of oxygen-containing functional groups on GO.

## Data Availability

The original contributions presented in the study are included in the article/Supplementary Material, further inquiries can be directed to the corresponding authors.
